# The Course of Obstructive Sleep Apnea Syndrome in Patients With Acromegaly During Treatment

**DOI:** 10.1210/clinem/dgz050

**Published:** 2019-10-15

**Authors:** Thalijn L C Wolters, Sean H P P Roerink, Linda C A Drenthen, Jolanda H G M van Haren-Willems, Margaretha A E M Wagenmakers, Johannes W A Smit, Adrianus R M M Hermus, Romana T Netea-Maier

**Affiliations:** 1 Department of Internal Medicine, Division of Endocrinology, Radboud University Medical Center, Nijmegen, The Netherlands GA; 2 Department of Pulmonary Diseases, Radboud University Medical Center, Nijmegen, The Netherlands GA; 3 Department of Internal Medicine, Center for Lysosomal and Metabolic Diseases, Erasmus MC University Medical Center Rotterdam, Rotterdam, The Netherlands GD

**Keywords:** acromegaly, sleep apnea syndrome, RDI, ODI, Epworth Sleepiness Scale, IGF-1

## Abstract

**Background:**

Obstructive sleep apnea syndrome (OSAS) is common in active acromegaly and negatively influences quality of life, morbidity, and mortality. This prospective study with 3 predetermined timepoints and a standardized treatment protocol investigates changes in sleep parameters during the first 2.5 years of acromegaly treatment.

**Methods:**

Before initiation of acromegaly treatment (medical pretreatment followed by surgery), polysomnography (PSG) was performed in 27 consecutive patients with treatment-naive acromegaly. PSG was repeated after 1 year (N = 24) and 2.5 years (N = 23), and anthropometric and biochemical parameters were obtained.

**Results:**

At baseline, 74.1% of the patients was diagnosed with OSAS. The respiratory disturbance index (RDI; *P =* 0.001), oxygen desaturation index (ODI; *P =* 0.001), lowest oxygen saturation (LSaO_2_; *P =* 0.007) and the Epworth Sleepiness Scale (ESS; *P* < 0.001) improved significantly during treatment, with the greatest improvement in the first year. After 2.5 years of treatment, all patients had controlled acromegaly. Of the 16 patients with repeated PSG and OSAS at baseline, 11 (68.8%) were cured of OSAS. Changes in RDI, ODI, LSaO_2_, and ESS correlated with insulin-like growth factor 1 levels.

**Conclusion:**

OSAS has a high prevalence in active acromegaly. There is a substantial decrease in prevalence and severity of OSAS following acromegaly treatment, with the largest improvement during the first year. Most patients recover from OSAS following surgical or biochemical control of the acromegaly. Therefore, a PSG is advised after diagnosis of acromegaly. When OSAS is present, it should be treated and PSG should be repeated during acromegaly treatment.

Acromegaly is a rare disease that is characterized by uncontrolled growth hormone (GH) secretion, most commonly caused by a pituitary adenoma, which leads to excessive production of insulin-like growth factor 1 (IGF-1) (1, 2). GH and IGF-1 hypersecretion lead to craniofacial abnormalities and tissue growth in the upper respiratory tract. These changes result in an increased incidence of—predominantly obstructive—sleep apnea syndrome ([O]SAS) (2–4), which is characterized by episodes of reduction or cessation of airflow during sleep. This leads to signs of disturbed sleep, such as snoring, restlessness, or resuscitative snorts.

In the general population, disturbed sleep is associated with poor neurocognitive performance (5) and an array of daytime symptoms, such as sleepiness, fatigue, and poor concentration (6, 7). Consequently, SAS severely influences the quality of life (8, 9). OSAS is associated with endothelial dysfunction, systemic inflammation, and dysglycemia, which are factors that are associated with the development of cardiovascular disease (CVD) (10). In addition, severe untreated OSAS is associated with increased all-cause and cardiovascular morbidity and mortality (11).

In acromegaly, the reported prevalence of SAS ranges from 44% to 87.5% among patients with active disease and from 35% to 58% in patients with controlled disease (12). However, previous prospective studies on SAS in acromegaly patients included only 2 time points and reports on the course of SAS after treatment of acromegaly are conflicting (3, 4, 9, 12–20): both persistence of SAS in patients with controlled disease and reversion of SAS in patients with ongoing acromegaly have been reported. Due to these heterogeneous outcomes—which may be caused by inclusion of treated patients at baseline, different durations of follow-up, unclear definitions of remission, and varying treatment regimens—the influence of acromegaly treatment on the course of SAS is still unclear. In order to overcome the aforementioned limitations and to gain knowledge on the effects of acromegaly treatment on SAS, as well as to improve SAS treatment and patient counseling, we systematically assessed the prevalence of SAS in consecutive treatment-naive patients with acromegaly and analyzed the influence of acromegaly treatment on SAS during the first 2.5 years after initiation of treatment, at 3 predetermined time points.

## Materials and Methods

### Particpants

All untreated adult patients with acromegaly who visited the outpatient department of the Radboudumc between July 2012 and June 2016 were eligible to be included in this study. The diagnosis of acromegaly was biochemically confirmed by an increased serum IGF-1 level (> 2 SD above the sex- and age-adjusted mean) and insufficient suppression of serum GH levels during an oral glucose tolerance test (oGTT; GH ≥ 0.4 µg/L) (1). Magnetic resonance imaging (MRI) of the pituitary gland was performed in each patient to identify a pituitary adenoma. Patients with comorbidities that could influence the prevalence of sleep apnea (eg, muscular dystrophy, unsubstituted hypothyroidism) were not eligible.

Patients visited our hospital at diagnosis (T_0_), after 1 year (T_1_), and after 2.5 years (T_2_). At each visit, patients underwent PSG, filled out the Epworth Sleepiness Scale, and were assessed for body weight and blood pressure. Measurement of IGF-1 level was performed on venous blood from the patient in the non-fasted state. At T_0_, height and GH levels were also measured.

After diagnosis, standard care consisted of pretreatment with a long-acting somatostatin receptor analogue (SSA) for approximately 6 months, followed by endoscopic endonasal transsphenoidal adenomectomy (EETA), or primary medical therapy in patients who were not suitable for surgery. If biochemical control was not obtained by SSA monotherapy, the GH-receptor antagonist pegvisomant (PEGV) or a dopamine agonist (DA) was added.

In the case of residual or recurrent disease after surgery, medical therapy was restarted postoperatively. When possible, patients underwent a second surgical approach. In the case of persistent IGF-1 levels above the reference range despite maximal tolerable medical therapy, patients underwent radiotherapy.

*Surgical control* was defined as postoperative IGF-1 levels within the sex- and age-adjusted reference range, combined with a random GH level < 1 µg/L or a sufficient suppression of serum GH levels (GH < 0.4 µg/L) during an oGTT, performed approximately 4 months after surgery (1, 21), without use of medication. *Biochemical control* was defined as IGF-1 levels within the sex- and age-adjusted reference range (21) with use of medication (SSA, PEGV, and/or a DA).

*Adrenal insufficiency* was defined as a morning serum cortisol < 100 nmol/L without use of glucocorticoids for 24 h, or a maximal cortisol response < 550 nmol/L during an insulin tolerance test (ITT) (22). Subclinical adrenal insufficiency was defined as normal morning cortisol levels with an insufficient response (cortisol < 550 nmol/L) during an ITT. Women were defined as *postmenopausal* when gonadotrophin levels were in the postmenopausal range and/or when they were older than 55 years. In premenopausal women and men, *hypogonadism* was defined as estrogen or total testosterone levels below the reference range. *Hypothyroidism* was defined as free thyroxin (fT4) plasma levels < 8 pmol/L (institutional reference range 8–22 pmol/L).

*Hypertension* was defined as use of antihypertensive therapy because of a previous diagnosis of hypertension or at least 3 office measurements of a systolic blood pressure ≥ 140 mm Hg and/or a diastolic blood pressure ≥ 90 mm Hg on different days (23).

*Diabetes mellitus* was defined as use of glucose-lowering medication based on a previous diagnosis of diabetes mellitus or when fasting glucose levels were ≥ 7 mmol/L and/or random glucose levels were ≥ 11.1 mmol/L on initial examination and after re-measurement at another date (24).

*Dyslipidemia* was defined as use of a lipid-lowering drug or as levels of low-density lipoprotein-cholesterol ≥ 5 mmol/L, total cholesterol ≥ 7 mmol/L and/or triglycerides ≥ 2 mmol/L at 2 or more measurements (25).

This study was conducted in accordance with the Declaration of Helsinki and approved by our local ethical committee (CMO regio Arnhem-Nijmegen; 2012–131). All subjects signed informed consent prior to participation.

### Hormone assays

Serum IGF-1 and GH levels were determined using a chemiluminescent immunometric assay (Liaison, DiaSorin, Saluggia, Italy).

### Polysomnography

Complete overnight polysomnography (PSG) was performed between 9:00 pm and 6:00 am using either a Compumedics System (Series E EEG/PSG, Compumedics Limited Global Corporate HQ, Victoria, Australia) or a SOMNOscreen™ plus PSG Tele + Video system (SOMNOmedics,GmbH, Randersacker, Germany). The results were analyzed according to the current standard of care (26) with Profusion PSG software (Compumedics; version 1.01 build 16) respectively Domino software (SOMNOmedics). Four-channel electroencephalography, electro-oculography, and chin electromyography were performed. Oronasal airflow was recorded by a thermistor and thoracic and abdominal respiratory efforts were measured by impedance plethysmography. Oxygen saturation was measured by finger pulse oximetry and electrocardiography was performed from standard leads. Body position was monitored by a position sensor. Patients who were treated by continuous positive airway pressure (CPAP) after diagnosis of SAS, discontinued this treatment at least 3 days prior to PSG at follow-up measurements. Respiratory events were scored according to the recommendations of the American Academy of Sleep Medicine (AASM) (6). An *apnea* was defined as an event of at least 10 seconds with an airflow drop by ≥ 90% compared to baseline during at least 90% of the event. *Obstructive* apneas are accompanied by continued inspiratory effort, which is absent in *central* apneas. *Mixed* apneas are characterized by absence of inspiratory effort in the initial part of the event, followed by resumption of inspiratory effort in the second part.

The *oxygen desaturation index* (ODI) is defined as ≥ 4% arterial oxygen desaturations per hour. *Hypopnea* was defined as an event of at least 10 seconds with an airflow drop by ≥ 30% compared to baseline, combined with a ≥ 4% desaturation from pre-event baseline values, during at least 90% of the event. *Respiratory effort–related arousals (RERAs)* are obstructive events with a sequence of breaths for at least 10 seconds, characterized by increased respiratory effort or by flattening of the inspiratory portion of the nasal pressure waveform, leading to an arousal from sleep. In addition, this sequence of breaths does not meet criteria for an apnea or hypopnea (26). The *respiratory disturbance index (RDI)* is the sum of the number of apneas, hypopneas and RERAs per hour of sleep during PSG. The *apnea hypopnea index (AHI)* is the sum of the number of apneas and hypopneas under these conditions.

The diagnosis of SAS was confirmed when the RDI was ≥ 5 combined with loud snoring, witnessed breathing interruptions, awakening due to gasping or choking and/or excessive daytime sleepiness (6). OSAS was defined mild when the RDI was ≥ 5 and < 15, moderate when ≥ 15 and < 30 and severe when RDI ≥ 30 (6). The term OSAS was used to describe both obstructive apneic and hypopneic events, since OSAS is the predominant form of SAS in patients with acromegaly and also in our cohort.

### Epworth Sleepiness Scale

The *Epworth Sleepiness Scale (ESS)* was used to measure daytime sleepiness (27). The ESS describes 8 situations, which are each scored on a scale from 0 to 3 based on the likeliness that the person would doze off or fall asleep in the specific situation (0 = would never doze; 1 = slight chance of dozing; 2 = moderate chance of dozing; 3 = high chance of dozing). A total ESS score from 0 to 10 is considered normal (28).

### Statistical analysis

Data were analyzed with SPSS 25.0. Data are represented as number with percentages for categorical variables and as mean with SD or as median with minimum and maximum values for continuous variables, depending on the normality of the distribution, which was tested by the Shapiro-Wilk test. At baseline, between-group differences in continuous variables were tested with an independent samples T-test for normally distributed and a Mann-Whitney U test for nonnormally distributed data. Fisher’s exact test was used for categorical variables. Spearman rank correlation was used to determine correlations. For correlations between a continuous and a dichotomous variable, point-biserial correlations (R_pb_) were determined.

All available prospective data were analyzed with a multilevel linear model or with Friedman’s two-way analysis, depending on the normality of the distribution. Nonnormally distributed data were log-transformed and the derived residuals were tested for normality. If log-transformation did not result in normally distributed residuals, nonparametric tests were used. The Hodges-Lehman test was used to determine median differences between measurements in nonparametric tests. For categorical values generalized linear models with likelihood ratios were used.

To calculate correlation coefficients on repeated observations within subjects, the method of Bland and Altman was used (29). All tests were two-tailed. *P* values of < 0.05 were considered statistically significant.

## Results

### Participant characteristics (Tables 1–4)

Thirty-two patients were eligible for participation; 5 patients refused because of time constraints (N = 2) or fear of the study procedures (N = 3). The remaining 27 patients were included in the study, of whom 12 (44.4%) were male. The mean age was 51.3 ± 13.5 years. Nineteen (70.4%) patients had a GH-secreting macroadenoma (> 1 cm), 7 patients had a microadenoma (≤ 1 cm) and 1 patient had a GH-releasing hormone–producing bronchial intermediate-grade neuroendocrine tumor (NET). In 1 patient, no oGTT was performed at diagnosis because of uncontrolled type 2 diabetes mellitus.

**Table 1.** Subgroup Comparison at Baseline (diagnosis; T_0_): Patients With and Without Obstructive Sleep Apnea Syndrome (OSAS)

Baseline (T_0_) Characteristics	Patients With OSAS (N = 20)	Patients Without OSAS (N = 7)	*P* Value
*Sex, male (%)*	11 (55)	1 (14.3)	0.09
*Age (years)*	49.7 ± 11.7	55.9 ± 17.9	0.42
*Weight (kg)*	95.3 ± 18.5	73.1 ± 16.5	0.01
*BMI (kg/m* ^*2*^)	29.7 ± 3.8	26.1 ± 4.1	0.07
*Duration of symptoms (years)*	8 (2–20)	6 (5–28)	0.74
*Diabetes mellitus (N, %)*	5 (25)	0 (0)	0.28
*Hypothyroidism (N, %)*	2 (10)	0 (0)	1
*Hypocortisolism (N, %)*	2 (10)	0 (0)	1
*Hypogonadism (N, %)*	7 (35)	1 (14.3)	0.63
*Random GH (µg/L)*	9.4 (2.4-32.6)	7.3 (1.8 -127.7)	0.28
*IGF-1 (nmol/L)*	106 ± 34.3	81.1 ± 31.1	0.1
*IGF-1 SDS*	7.8 (5.2-23.2)	5.6 (3.5-12.7)	0.08
*Current smoker (N, %)*	0 (0)	2 (28.6)	0.06
*Alcohol consumption: yes (N, %)*	15 (75)	3 (42.9)	0.18
*Macroadenoma (N, %)*	15 (75)	4 (57.1)	0.36
*Microadenoma (N, %)*	5 (25)	2 (28.6)	
*GHRH-producing NET (N, %)*	0 (0)	1 (14.3 )	
*AHI (/hour)*	19.8 (5.8-64.6)	2.9 (0.5-6.1)	<0.001
*RDI (/hour)*	27.9 (7.9-99.2)	5.2 (1.2-7.1)	<0.001
*ODI (/hour)*	19.8 (0.6-80.5)	4.1 (1.5-7.1)	0.002
*LSaO*_*2*_ *(%)*	81.3 ± 6.4	84.8 ± 5.3	0.2
*ESS*	11.3 (4.8)	12 (3.1)	0.78
*OSAS type (N, %)*			
* Obstructive*	20 (100)	NA	NA
* Central*	0 (0)		

Values are displayed as mean with (SD) or as median with minimum and maximum, depending on the normality of the distribution. Categorical variables are displayed as numbers (percentage). Abbreviations: AHI, apnea hypopnea index; BMI, body mass index in kg/m^2^; ESS, Epworth Sleepiness Scale; GH, growth hormone; GHRH, GH-releasing hormone; IGF-1, insulin-like growth factor 1; LSaO_2,_ lowest oxygen saturation; NA, not applicable; ODI, oxygen desaturation index; OSAS, obstructive sleep apnea syndrome; RDI, respiratory disturbance index; SDS, standard deviation score.

**Table 2.** Course of Clinical, Biochemical and Sleep Characteristics

Patient Characteristics	T_0_ (N = 27)	T_1_ (N = 24)	T_2_ (N = 23)	*P* Value ΔT_0_-T_1_	*P* Value ΔT_0_-T_2_
*Sex, male (%)*	12 (44.4)	12 (50)	10 (43.5)	**NA**	**NA**
*Age (years)*	51.3 ± 13.5	NA	NA	**NA**	**NA**
*Weight (kg)*	89.5 ± 20.3	93.6 ± 23	87.7 ± 19.5	**0.06**	**0.07**
*BMI (kg/m* ^*2*^)	28.7 ± 4.1	29.8 ± 5.6	28.3 ± 5	**0.07**	**0.07**
*Duration of symptoms (years)*	7.5 (2–28)	NA	NA	**NA**	**NA**
*Diabetes mellitus (N, %)*	5 (18.5)	5 (20.8)	5 (21.7)	**1**	**1**
*Hypothyroidism (N, %)*	2 (7.4)	3 (12.5)	3 (13)	**0.67**	**0.91**
*Hypocortisolism (N, %)*	2 (7.4)	2 (8.3)	2 (8.7)^a^	**0.9**	**0.99**
*Hypogonadism (N, %)*	8 (29.6)	6 (25)	5 (2.17)^a^	**0.53**	**0.87**
*Random GH (µg/L)*	7.7 (1.8–127.7)	NA	NA	**NA**	**NA**
*IGF-1 (nmol/L)*	99.5 ± 34.8	31.7 ± 16.3	23.5 ± 6.9	**<0.001**	**<0.001**
*IGF-1 SDS*	7.6 (3.5-23.2)	1.5 (-2; 4.8)	1 (-1.2; 2.3)	**<0.001**	**<0.001**
*Current smoker (N, %)*	2 (7.4)	2 (8.3)	3 (8.7)	**1**	**0.92**
*Alcohol consumer: yes (N, %)*	18 (66.7)	16 (66.7)	14 (60.9)	**1**	**0.9**
*Macroadenoma (N, %)*	19 (70.4)	NA	NA	**NA**	**NA**
*RDI (/hour)*	22.4 (1.2-99.2)	12.5 (0.2-95)	7.2 (0-24.1)	**0.18**	**0.001**
*AHI (/hour)*	14.2 (0.5-64.6)	5.3 (0-52.8)	4 (0-23.6)	**0.039**	**0.001**
*ODI (/hour)*	12.8 (0.6-80.5)	7.0 (0.1-62.7)	3.5 (0-21.1)	**0.01**	**0.001**
*LSaO*_*2*_ *(%)*	84 (68-90)	86 (67-92)	87 (72-93)	**0.05**	**0.007**
*ESS*	11.2 ± 4.4	6.8 ± 3	6.6 ± 3.3	**<0.001**	**<0.001**
*Apnea type* ^b^ *(N, %)*				**0.91**	**0.15**
* Obstructive/hypopnea*	26 (96.3)	23 (95.8)	22 (95.7)		
* Mixed (obstructive + central)*	1 (3.7)	0	0		
*OSAS severity (based on RDI; N, %)*				**0.001**	**<0.001**
* No (<5)*	7 (25.9)	11 (45.8)	18 (78.3)		
* Mild (5-14)*	5 (18.5)	8 (33.3)	3 (13.0)		
* Moderate (15-29)*	6 (22.2)	3 (12.5)	2 (8.7)		
* Severe (>30)*	9 (33.3)	2 (8.3)	0 (0)		
*Disease status (N, %)*				**<0.001**	**<0.001**
* Untreated/Active*	27	0	0		
* Surgical remission*	0 (0)	15 (62.5)	14 (60.9)		
* Biochemical remission*	0 (0)	2 (8.3)	9 (39.1)		
* Active despite treatment*	0 (0)	7 (29.2)	0 (0)		

Values are displayed as mean with standard deviation (SD) or as median with minimum and maximum, depending on the normality of the distribution. Categorical variables are displayed as numbers (percentage). Abbreviations: AHI, apnea hypopnea index; BMI, body mass index in kg/m^2^; ESS, Epworth Sleepiness Scale; GH, growth hormone; IGF-1, insulin-like growth factor 1; LSaO_2,_ lowest oxygen saturation; NA, not applicable; ODI, oxygen desaturation index; OSAS, obstructive sleep apnea syndrome; RDI, respiratory disturbance index; SDS, standard deviation score. ^a^One male with hypogonadism and hypocortisolism did not undergo a PSG at T_2_. ^b^One patient did not demonstrate any event during PSG at T_1_ and T_2_.

**Table 3.** Course of Treatment, RDI, OSAS, BMI, and IGF-1 Levels in Individual Patients

Course of Individual Patients				Baseline—T_0_				1 Year—T_1_				2.5 Years—T_2_		
No	Sex	Treatment	RDI	OSAS	BMI	IGF-1	RDI	OSAS	BMI	IGF-1	RDI	OSAS	BMI	IGF-1
1	M	MS (0-6) + S: cured	46.3	+	31.1	208	17.9	+	29.4	O	14.2	-	30.7	13
2	M	MS (0-6) + S: cured	24.2	+	24.9	80.7	3.8	-	25.6	34.8	5.1	-	24	28
3	M	MS (0-6) + S: cured	35.2	+	25.6	111	6.6	+	24.8	O	5.6	-	24.7	29.8
4	M	MS (0-7) + S: cured	54.6	+	24.6	61.9	95	+	25	25.1	8.4	+	24.8	29.4
5	M	MS (0-6) + S: cured	26.9	+	28.1	66.7	11.6	+	28	8.5	14.1	-	28.7	34.1
6	F	MS (0-6) + S: cured	32.5	+	23.5	108	14.1	+	23.1	23.6	7.9	-	22.5	18.2
7	F	MS (0-6) + S: cured	17.6	+	30.7	77.2	6.1	-	30.5	20.4	0.4	-	31.5	22.4
8	F	MS (0-6) + S: cured	27.9	+	27.5	97.3	15.7	+	34.6	23.5	1.2	-	28.5	25.5
9	F	MS (0-6) + S: RD	12.1	+	32.3	79.8	3	-	33.3	36.5	1.3	-	31.4	28.3
		MS (14-30): BC (16-30)												
10	F	MS (0-8) + S: cured	99.2	+	32.6	80.1	3.9	-	35.5	19.3	5.9	-	35	10.2
11	M	S (2): cured	5.5	-	28.7	122.1	13.4	-	25.5	34.2	7.2	-	26.1	30.4
12	F	Primary MS (0-30): BC (6-30)	6.2	-	25.7	40.6	1.9	-	26.5	24.7	14.4	-	26.8	28
13	F	MS (0-6) + S: RD	1.2	-	21.2	83.7	4	-	21.7	48.3	0	-	20.1	17.8
		MS (13-30): BC (15-30)												
14	F	MS (0-6) + S: cured	7.1	-	21.3	63.8	18.1	+	21.2	11	4.6	-	22	11.7
15	F	MS (0-6) + S: RD	3.1	-	32.6	118.7	3.7	-	37.5	54.4	1.3	-	38.4	25.8
		MS (12-24) + MP (15-22) + reS (24): cured												
16	M	MS (0-6) + S: RD	77.4	+	26.4	109.5	70.3	+	28.2	27.4	24.1	+	26.7	20.3
		MS (11-30): BC (12-30)												
17	F	MS (0-6) S: RD	20.9	+	29.1	146	O	O	27.6	83.2	9.7	-	28	25.9
		MS (8-30) + MP (11-30) + SRT (20-21): BC (29-30)												
18	M	S (1.5): RD	35.9	+	30.2	132.5	13.9	+	29.7	40.7	8.6	-	29.1	28
		MS (2.5-30): BC (14-30)												
19	F	MS (0-11) + S: cured	2.2	-	25.8	53.5	2.6	-	26.3	17.2	9.1	-	27	15.6
20	F	MS (0-6) + S: RD	5.2	-	27.5	85.2	O	O	24.6	20.2	2.4	-	25	21.1
		MS (8-30): BC (10-30)												
21	M	MS + MD (0-6) + S: RD	O^a^	+	36.2	95.7	3.6	-	38.4	34.9	23.7	+	30.3	34
		MS + MD (14-30): BC (24-30)												
22	M	MS (0-6) + MP (4-6) + S: RD	13.3	+	33.1	114.2	0.2	-	34.3	45.9	O	O	30.4	27
		MS (9-30) + MP (10-30) + GRS (12): BC (15-30)												
23	M	MS (0-6) + S: cured	28	+	31.6	130.4	35.4	+	30.5	40.3	24.1	+	28.8	21.9
24	F	MS (0-6) + S: cured	23.8	+	25.6	105.8	26.7	+	26.9	31.6	O	O	26.4	28.7
25	M	MS (0-5) + S: cured	47.3	+	34.3	145.4	16.1	+	35	20.6	O	O	31.1	33
26	F	Lost to follow-up	7.9	+	30.8	84.5	Lost to follow-up				Lost to follow-up			
27	F	Primary MS (0-30) + MP (9-30): BC (22-30)	15.2	+	35.1	84.6	24.3	+	43.1	36.1	5.2	+	41	19.3
		**Patients with OSAS**		**20 (74.1%)**				**13 (54.2%)**				**5 (21.7%)**		

In patients with medical pretreatment, surgery and cessation of medical treatment took place simultaneously. The moment or timespan of treatment in months is displayed between parentheses. In the OSAS column, a ‘+’ means that OSAS is present, a ‘-’ means that OSAS is absent. Abbreviations: O, data not available; M, medical treatment; MS, medical treatment with a somatostatin analogue (SSA); MP, medical treatment with pegvisomant (PEGV); MD, medical treatment with a dopamine agonist; MS + MP, medical treatment with SSA combined with PEGV; MS + MD, medical treatment SSA combined with a dopamine agonist; S, surgery; SRT: stereotactic radiotherapy; GRS, gammaknife radiosurgery; BC, biochemical control with use of medication; Re-s, second surgical procedure; RD, residual or recurrent disease; AHI, apnea hypopnea index; BMI, body mass index in kg/m^2^; ESS, Epworth Sleepiness Scale; GH, growth hormone; IGF-1, insulin-like growth factor 1; LSaO_2,_ lowest oxygen saturation; NA, not applicable; ODI, oxygen desaturation index; OSAS, obstructive sleep apnea syndrome; RDI, respiratory disturbance index. ^a^The baseline PSG of this patient was of insufficient quality to calculate RDI or AHI, but he had an oxygen desaturation index (ODI) of 80/hour and has been diagnosed with severe OSAS prior to study participation.

**Table 4.** Course of Clinical, Biochemical, and Sleep Characteristics in Patients Who Underwent PSG at T_0_ and T_2_

Patient Characteristics	T_0_ (N = 23)	T_1_ (N = 21)	T_2_ (N = 23)	*P* Value ΔT_0_-T_1_	*P* Value ΔT_0_-T_2_
*Sex, male (%)*	10 (43.5)	10 (47.6)	10 (43.5)	**NA**	**NA**
*Age (years)*	52.61 ± 13.4	NA	NA	**NA**	**NA**
*Weight (kg)*	88.1 ± 19.6	90.4 ± 22.2	87.7 ± 19.5	**0.1**	**0.11**
*BMI (kg/m* ^*2*^)	28.4 ± 4.1	29.2 ± 5.6	28.3 ± 5	**0.1**	**0.11**
*Duration of symptoms (years)*	7 (2-28)	NA	NA	**NA**	**NA**
*Diabetes mellitus (N, %)*	5 (21.7)	5 (23.8)	5 (21.7)	**1**	**1**
*Hypothyroidism (N, %)*	2 (8.7)	3 (14.3)	3 (13)	**0.66**	**0.83**
*Hypocortisolism (N, %)*	1 (4.3)	1 (4.8)	2 (8.7)	**1**	**0.8**
*Hypogonadism (N, %)*	6 (26.1)	5 (23.8)	5 (21.7)	**1**	**0.94**
*Random GH (µg/L)*	24.2 (5.3-383)	NA	NA	**NA**	**NA**
*IGF-1 (nmol/L)*	97.3 ± 36.1	31.6 ± 27.4	23.5 ± 6.9	**<0.001**	**<0.001**
*IGF-1 SDS*	7.6 (3.5-23.2)	1.5 (-2; 9.1)	1 (-1.2; 2.3)	**<0.001**	**<0.001**
*Current smoker (N, %)*	2 (8.7)	2 (9.5)	3 (13)	**1**	**0.88**
*Alcohol consumer: yes (N, %)*	15 (65.2)	13 (61.9)	14 (60.9)	**1**	**0.95**
*Macroadenoma (N, %)*	17 (73.9)	NA	NA	**NA**	**NA**
*RDI (/hour)*	22.6 (1.2-99.2)	11.6 (1.9-95)	7.2 (0-24.1)	**0.057**	**0.002**
*AHI (/hour)*	15.4 (0.5-64.6)	5.2 (1.1-52.8)	4 (0-23.6)	**0.046**	**0.002**
*ODI (/hour)*	15.2 (1.5-80.5)	6.5 (1.4-62.7)	3.5 (0-21.1)	**0.01**	**<0.001**
*LSaO*_*2*_ *(%)*	84 (68-90)	86 (67-91)	87 (72-93)	**0.15**	**0.013**
*ESS*	11.7 ± 4.3	7 ± 3.2	6.6 ± 3.3	**<0.001**	**<0.001**
*Apnea type* ^a^ *(N, %)*				**1**	**1**
*Obstructive/hypopnea*	22 (95.7)	21 (100)	22 (95.7)		
*Mixed (obstructive + central)*	1 (4.3)	0	0		
*OSAS severity (based on RDI; N, %)*				**0.04**	**<0.001**
* No (<5)*	4 (17.4)	10 (47.6)	18 (78.3)		
* Mild (5-14)*	6 (26.1)	6 (28.6)	3 (13)		
* Moderate (15-29)*	5 (21.7)	3 (14.3)	2 (8.7)		
* Severe (>30)*	8 (39.1)	2 (9.5)	0 (0)		
*Disease status (N, %)*					
* Untreated/Active*	23 (100)	0 (0)	0 (0)		
* Surgical remission*	0 (0)	13 (61.9)	14 (60.9)	**<0.001**	**<0.001**
* Biochemical remission*	0 (0)	2 (9.5)	9 (39.1)		
* Active despite treatment*	0 (0)	6 (28.6)	0 (0)		

Values are displayed as mean with (SD) or as median with minimum and maximum, depending on the normality of the distribution. Categorical variables are displayed as numbers (percentage). Abbreviations: AHI, apnea hypopnea index; BMI, body mass index in kg/m^2^; ESS, Epworth Sleepiness Scale; GH, growth hormone; IGF-1, insulin-like growth factor 1; LSaO_2,_ lowest oxygen saturation; NA, not applicable; ODI, oxygen desaturation index; OSAS, obstructive sleep apnea syndrome; RDI, respiratory disturbance index; SDS, standard deviation score. ^a^One patient did not demonstrate any event during PSG at T_1_ and T_2_.

One patient completed his biochemical female-to-male gender transformation (as reported in a case report (30)) 20 months prior to baseline and is classified as male in the data analysis.

One patient discontinued the study shortly after T_0_ because she moved to another city and continued her treatment in another hospital. Twenty-six patients completed the follow-up period.

#### Disease control and acromegaly treatment

 (Table 3**).** Twenty-three patients (85.2%) started pretreatment with a SSA, for a mean duration of 6 months (range, 5–11), followed by EETA. In 1 of these patients, PEGV was added to the pretreatment because of insufficiently-controlled IGF-1 levels with SSA monotherapy. A DA was added in another patient because of concomitant hyperprolactinemia. One patient refused SSA pretreatment and underwent EETA 6 weeks after baseline. Two women did not undergo EETA, due to advanced age and the presence of an inoperable giant adenoma. They were primarily treated with a SSA and a SSA combined with PEGV, respectively. The patient with the bronchial NET underwent a partial lobectomy without pretreatment 8 weeks after diagnosis.

At T_1_, 15 of the 24 surgically treated patients (62.5%) were in surgical control and 9 patients (37.5%) had residual or recurrent disease. Four patients had repeatedly normal IGF-1 levels combined with a mildly disturbed oGTT (GH nadir 0.7–0.8 µg/L). Since their IGF-1 values fell within the reference range, they were considered surgically controlled patients in the analysis (1).

Of the patients who underwent PSG (N = 24) at T_1_, 15 patients (62.5%) were surgically controlled, 2 patients (8.3%) were biochemically controlled and 7 patients (29.2%) had active acromegaly despite treatment. Six patients were treated with SSA monotherapy, 1 patient with a SSA combined with a DA, and 2 patients with a SSA combined with PEGV.

At T_2_, all 23 patients who underwent PSG were in surgical (N = 14; 60.9%) or biochemical control (N = 9; 39.1%). Five patients were treated with SSA monotherapy, 1 patient with a SSA combined with a DA, and 3 patients with a SSA combined with PEGV.

Between T_1_ and T_2_, 1 female participant underwent a second surgical procedure and was in surgical remission afterwards. In addition, 1 male patient underwent gammaknife radiosurgery and 1 female patient underwent stereotactic radiotherapy between T_1_ and T_2_ while they were being treated with a SSA and PEGV.

Between T_0_ and T_2_, IGF-1 levels decreased from 99.5 ± 34.8 to 23.5 ± 6.9 nmol/L (*P* < 0.001); no significant changes in body mass index (BMI) were found between these time points.

#### Hormonal deficiencies.

At baseline, 2 patients had a history of hypothyroidism, 1 primary and 1 secondary, and had been adequately treated with substitution therapy for at least 3 months. One patient developed hypothyroidism due to autoimmune thyroiditis (anti-TPO levels > 1000 U/mL) during treatment with SSA; at T_1_, all 3 patients were adequately substituted with levothyroxine for more than 3 months.

From diagnosis until the end of the study, 1 patient had secondary adrenal insufficiency and received daily glucocorticoid substitution therapy. Another subject had normal unstimulated cortisol levels but failed to reach the maximal cortisol response (ie, serum cortisol level > 550 nmol/L) during an ITT both before and after EETA. Therefore, he only used substitution during physical or mental stress. One female developed a subclinical adrenal insufficiency after a second surgical procedure between T_1_ and T_2_.

At T_0_, 8 men had hypogonadism: 1 had unsubstituted primary hypogonadism as a result of a bilateral orchidopexia in childhood, and the other 7 men had unsubstituted secondary hypogonadism. Eleven women were postmenopausal. At T_2_, 3 men had recovered from secondary hypogonadism. Four hypogonadal men were substituted with a stable dose of testosterone for at least 3 months and 1 male with mild and asymptomatic hypogonadism refused substitution therapy. One premenopausal woman developed secondary amenorrhea combined with estrogen values below the reference range after postoperative radiation therapy between T_1_ and T_2_.

### Polysomnography (PSG) and Epworth Sleepiness Scale (ESS) (Figs. 1 & 2)

**Figure 1.**
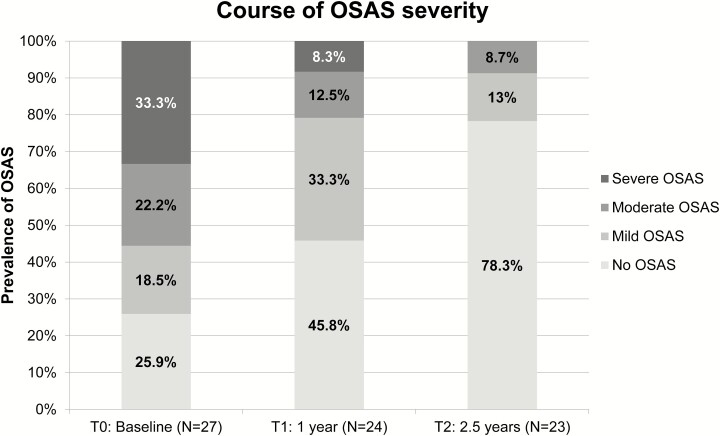
Course of obstructive sleep apnea syndrome (OSAS) severity per time point.

PSG at T_0_ was performed in 27 patients, of whom 20 were diagnosed with OSAS (74.1%). Five patients had mild, 6 moderate, and 9 severe OSAS (Fig. 1; Table 2). Patients with OSAS had a higher body weight (95.3 vs 73.1 kg; *P =* 0.01) than patients without OSAS. BMI was higher, but not statistically significantly, in patients with OSAS relative to those without OSAS (29.7 vs 26.1 kg/m^2^; *P =* 0.07). ESS scores did not differ between patients with or without OSAS (11.3 vs 12; *P =* 0.78). However, ESS scores in patients with no or mild OSAS were lower compared with patients with moderate or severe OSAS (9.33 vs 12.8; *P =* 0.04). In addition, IGF-1, but not GH, levels were lower in patients with no or mild OSAS compared with patients with moderate or severe OSAS (83.3 vs 112.5 nmol/L; *P =* 0.02). Eleven patients had already used or started CPAP based on the PSG at T_0_. The 5 patients with mild OSAS were not treated with CPAP. One patient chose mandibular repositioning appliance treatment and 3 patients refused OSAS treatment despite having moderate OSAS.

**Figure 2.**
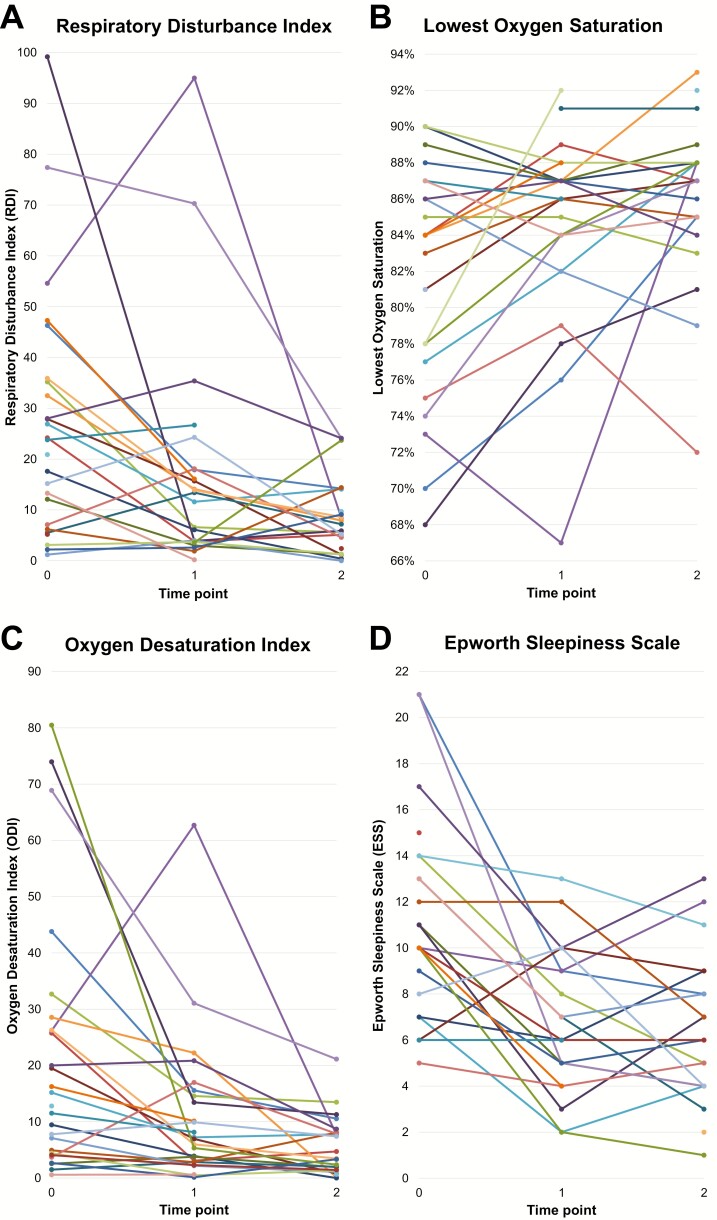
Course of sleep parameters and ESS. (A) Respiratory Disturbance Index (RDI); (B) Lowest oxygen saturation; (C) Oxygen Desaturation Index (ODI); (D) Epworth Sleepiness Scale (ESS). Each colored line represents the course of the parameter in a single subject.

PSG was repeated 1 year after diagnosis (T_1_) in 24 patients, and 2.5 years after diagnosis (T_2_) in 23 patients. At T_1_, 1 patient was lost to follow up, and 2 PSGs were of insufficient quality due to technical problems and were omitted from the data analysis. Of these patients, 2 had OSAS at baseline (patients 17 and 26) and 1 (patient 20) did not have OSAS. Of the 20 patients who had OSAS at T_0,_ 6 (30%) were cured of OSAS at T_1_ (Table 3). Of the remaining patients, 2 had severe, 3 moderate, and 7 mild OSAS (Table 2; Fig. 1). In addition, 1 patient developed mild OSAS during follow-up. Sleep parameters worsened in 5 patients (20.8%; 3 male and 2 female) between T_0_ and T_1_, although there was no increase in IGF-1 levels in these patients (4 out of 5 had a normal or decreased IGF-1 level relative to T_0_), they did not develop pituitary hormonal deficiencies, and BMI was stable or declined in all but 1 female patient, who had a significantly higher BMI at T_1_ compared to T_0_. The remaining 5 patients did not have OSAS at T_0_ nor at T_1_. Of the 11 patients using CPAP, 5 (45.5%) successfully discontinued CPAP therapy at T_1_ due to improvement of OSAS.

At T_2_, 3 patients were not willing to undergo PSG due to time constraints; 1 of whom was cured of OSAS at T_1_ while the other 2 had mild OSAS at T_1_, for which 1 used CPAP therapy during the entire follow-up period. At T_2,_ 11 of the 16 patients with OSAS at T_0_ and repeated PSGs were cured of OSAS (68.8%). In all other patients, OSAS severity was strongly reduced: 2 patients had moderate and 3 patients had mild OSAS (Table 2; Fig. 1). Regarding the total group with OSAS at baseline (N = 20), cure of OSAS was established in 12 patients during follow-up (60%) and OSAS persisted in 5 patients. In 3 patients, who did not undergo PSG at T_2_, the presence of absence of OSAS at T_2_ was unknown. In Table 4, the parameters displayed in Table 2 are depicted for the 23 patients who underwent PSG at both T_0_ and T_2_.

Two patients (1 male with OSAS, 1 female without OSAS) had a deterioration of sleep parameters between T_1_ and T_2_ without changes in IGF-1 levels, presence of pituitary hormonal deficiencies, and with a similar BMI in 1 patient and a lower BMI in the other patient.

The patient who developed mild OSAS at T_1_ was cured from OSAS at T_2_; the other 4 patients who had a deterioration of sleep parameters at T_1_ also showed an improvement of sleep parameters at T_2_.

The prevalence of established OSAS was 21.7% (5 of 23 patients) at T_2_. CPAP was discontinued in 2 additional patients at T_2_. In total, CPAP therapy was ended because of amelioration of OSAS in 7 out of 11 (63.6%) patients who had been using CPAP since T_0_.

The RDI, ODI, and ESS scores decreased between T_0_ and T_2_ (*P =* 0.001, *P =* 0.001, and *P* < 0.001, respectively), whereas the LSaO_2_ that was measured during PSG increased (*P =* 0.007); the largest changes were observed between T_0_ and T_1_ (Fig. 2; Tables 2 and 3).

### Correlation analysis

The correlation between RDI and AHI was 0.94 (*P* < 0.001); the RDI and AHI correlated with the ODI (both R 0.87; *P* < 0.001). Male gender correlated with the RDI and AHI (R_pb_ 0.47; *P =* 0.012 and R_pb_ 0.49; *P =* 0.009, respectively), and with the ODI (R_pb_ 0.46; *P =* 0.016). At baseline, GH levels correlated with the ODI (R 0.39; *P =* 0.049) and ESS scores (R 0.5; *P =* 0.01), but not with other sleep parameters. None of the sleep parameters correlated with BMI or body weight after correction for gender.

At baseline, OSAS severity correlated with IGF-1 levels (R 0.38; *P =* 0.049), the RDI (R 0.95; *P* < 0.001), ODI (R 0.92; *P* < 0.001), LSaO_2_ (R-0.56; *P =* 0.003), and male gender (R_pb_ 0.59; *P =* 0.001). OSAS severity did not correlate with the presence of hormonal deficiencies, ESS scores, or GH levels. Using Blant-Altman correlations for repeated measures within individuals, the decrease in IGF-1 levels during follow-up correlated with improvement in RDI (R 0.51; *P* < 0.001), AHI (R 0.53; *P* < 0.001), ESS (R 0.70; *P* < 0.001), and ODI (R 0.53; *P* < 0.001). The LSaO_2_ negatively correlated to the decrease in IGF-1 levels (R −0.43; *P =* 0.003). The decrease of ESS scores correlated with the improvement in RDI (R 0.4; *P =* 0.01), AHI (R 0.39; *P =* 0.01), ODI (R 0.56; *P* < 0.001), and LSaO_2_ (R −0.44; *P =* 0.004). To conclude, the decrease in IGF-1 levels is associated with a decrease in RDI/AHI, ODI, and ESS and an increase in LSaO_2_ within a patient over time.

## Discussion

This is the first prospective study that systematically assessed the presence and course of OSAS in consecutive treatment-naive patients with acromegaly, from diagnosis until 2.5 years after start of treatment. We found that, when performing screening for OSAS at diagnosis, 74.1% of the patients with untreated acromegaly had OSAS. Successful treatment of acromegaly resulted in a substantial reduction in the prevalence and severity of OSAS. The greatest improvement in OSAS severity and prevalence occurred in the first year after initiating treatment, and a strong correlation between change in sleep parameters and IGF-1 levels was observed.

The innovative and distinctive aspects of our study are its homogeneous cohort of consecutive treatment-naive patients, its standardized treatment protocol, and the systematical assessments at 3 predetermined time points during a relatively long follow-up of 2.5 years. In addition, inclusion of only treatment-naive patients excluded treatment-related effects on sleep parameters at baseline. A prospective study with this size and study protocol has not been conducted before.

The prevalence of OSAS that we observed in untreated acromegaly patients corresponds with the previous reported prevalence of OSAS in active acromegaly (44%–87.5%) (3, 12, 14, 17–19), whereas the prevalence of OSAS in controlled patients (21.7%) was lower compared to previous reports (35%–58%) (3, 17–19, 31).

In addition, OSAS severity diminished in all subjects during acromegaly treatment, which is in line with previous studies, which reported that both SSA treatment (7, 32, 33) and adenomectomy (34) are effective in attenuating OSAS severity in acromegaly, even in patients without completely controlled acromegaly (16).

Although previous studies had already indicated the high prevalence of OSAS in acromegaly patients and the potential for OSAS improvement after remission of acromegaly, the findings of the present longitudinal study are highly relevant since they provide more precise information about the effects of acromegaly treatment on the course of OSAS. This information highlights the necessity of disease control and could have a large impact on patient counseling and management of OSAS, and thereby quality of life, in acromegaly patients.

The differences in OSAS prevalence in controlled patients between the current study and previous studies can probably be explained by the fact that our longitudinal study had a predetermined follow-up period and consisted of 3 measurements at predetermined intervals. Previous prospective studies performed PSG at baseline and repeated PSG only once after variable periods of time (1–22 years after biochemical remission or 6 weeks to 6 months after surgery or start of medical treatment) (3, 17, 18, 33–35), which obscured an accurate assessment of the course of OSAS. Furthermore, we only included consecutive treatment-naive patients, whereas others also included patients with active acromegaly despite treatment (17–19, 32), as acromegaly treatment is known to reduce OSAS severity, even when IGF-1 levels are not normalized (7, 16, 32). Previous studies also often describe small and heterogeneous cohorts, or used different or unclear criteria for remission of acromegaly or OSAS (3, 9, 17–20, 31).

In this study, the RDI was used for diagnosing OSAS, since it largely determines OSAS severity and consequently the indication for CPAP therapy (6). However, this most likely does not explain the difference in OSAS prevalence with previous studies using the AHI (3, 16, 19, 32), since the majority of our patients with OSAS also fulfilled the criteria for OSAS based on the AHI (95% at T_0_ and 100% at T_2_), and a strong correlation between the RDI and AHI (R 0.96; *P* < 0.001) was observed.

A recent study found that patients with serum IGF-1 levels in the highest quartiles were more likely to have OSAS (12). Although a strong trend towards higher absolute IGF-1 levels (106 vs 81.1 nmol/L; *P =* 0.1) and IGF-1 standard deviation score (7.8 vs 5.6; *P =* 0.08) was observed in OSAS patients at baseline, this difference was not statistically significant. As reported by some (14, 18, 19), but not all authors (3, 17, 20), OSAS severity at baseline did correlate with IGF-1 levels. In addition, when comparing patients with no or mild OSAS vs patients with moderate or severe OSAS, IGF-1 levels were lower in patients with no/mild OSAS. We also observed a strong correlation between the changes in the RDI (and AHI) and IGF-1 levels in time, which has not been reported earlier in prospective studies, although a correlation between GH levels and the AHI was mentioned (33). However, previous studies were restricted by variable intervals between PSG, and by small and heterogeneous cohorts, which may have hampered correlation analysis.

Most likely, soft tissue hypertrophy and airway narrowing are major causal factors for the development of OSAS in active acromegaly (3, 4). Uvula and tongue hypertrophy (3, 7, 33), mucosal thickening/edema of the upper airways and bronchi (18), and increased collapsibility of the passive pharynx (36) are a result of GH and IGF-1 hypersecretion (37, 38) and are reported to be related to the AHI (33, 39).

Moreover, upper airway mucosal edema is an inflammatory process, and IGF-1 excess has been suggested to be a pro-inflammatory state (40, 41). Therefore, lowering of IGF-1 levels by acromegaly treatment may lead to reversal of these pro-inflammatory changes (42) and—consequently—soft tissue swelling, and thereby impact on OSAS severity (43). Indeed, acromegaly treatment resulting in IGF-1 normalization is reported to reduce the RDI/AHI and tongue volume, and IGF-1 levels correlated with tongue volume (7, 44).

Importantly, next to a strong reduction in the RDI, the LSaO_2_ also increased from 84% to 87% between T_0_ to T_2_. This is clinically relevant since hypoxemia, reflected by the LsaO_2_, is suggested to play a key role in the development of OSAS-associated CVD (45, 46).

Besides objective sleep parameters, ESS scores—as a subjective measure of sleepiness—correlated with GH and IGF-1 levels at baseline, and with IGF-1 levels over time. Over time, the improvement of ESS scores correlated with the improvements in the RDI and AHI, in line with a previous report (47). Importantly, CPAP therapy may have modified ESS scores at T_1_ and T_2_ in OSAS patients, since CPAP reduces sleepiness in the majority of patients (48).

In addition, ESS scores did not reliably distinguish between patients with and without OSAS at baseline. This can be explained by the low sensitivity, and moderate specificity and accuracy of the ESS in the identification of patients with OSAS (49).

Probably, additional risk factors other than acromegaly *per se* exist for developing OSAS in acromegaly, since patients with OSAS tended to have a higher body weight and BMI, and were predominantly male. These classical risk factors for OSAS are reported both in patients with acromegaly (7, 12, 16, 18, 50, 51) and in OSAS patients from the general population (8, 52). In contrast to earlier reports in acromegaly (4, 7, 16, 18, 53), OSAS was not related to age or patient-reported disease duration.

Importantly, besides GH/IGF-1, other pituitary-derived hormones may also influence sleep parameters. Adrenal insufficiency has a prevalence of approximately 12% in acromegaly (54, 55). However, although both increased (56) and decreased (57) cortisol levels have been reported in OSAS patients, the majority of studies have not demonstrated a relation between presence of OSAS and cortisol levels (58). With respect to thyroid dysfunction, in general, the prevalence of hypothyroidism and hyperthyroidism was similar in OSAS patients and healthy controls (0.4%–5%) (59–61), but the prevalence of subclinical hypothyroidism (11%) was higher in OSAS patients (60, 62). Lower estradiol and/or progesterone levels are associated with the occurrence of sleep-disordered breathing (63). In addition, low testosterone levels are associated with an increased risk of OSAS (64) and short-term testosterone replacement is reported to worsen or induce OSAS (65), whereas others report no effects on OSAS severity (31). Hypogonadism has a high prevalence (53%–70%) in active acromegaly (66, 67), although the gonadal axis recovers after achievement of (biochemical) remission in a significant number of cases (67–69). In the present study, 3 (37.5%) patients with hypogonadism at diagnosis recovered after treatment, and substitution therapy was started in 5 patients. Since the prevalence of hormonal deficiencies was similar in patients with and without OSAS, and did not significantly correlate with sleep parameters, we did not correct for hormonal deficiencies in the statistical analysis.

Regarding the course of OSAS, the largest improvement of OSAS in our cohort was observed during the first year of treatment, although in most patients, the improvements in sleep parameters continued up to the end of follow-up. Interestingly, a deterioration of sleep parameters during acromegaly treatment was observed in 5 patients between T_0_ and T_1_ (followed by a subsequent improvement of sleep parameters between T_1_ and T_2_) and in 2 patients between T_1_ and T_2_, a phenomenon that has been reported earlier (14, 18, 32). There was no relation with BMI and IGF-1 levels in any but 1 patient (patient 27). Possibly, the deterioration of sleep parameters may be explained by variability in changes of soft tissue volume, or other factors we did not measure, during acromegaly treatment (14, 18).

Importantly, all but 2 patients no longer required CPAP therapy at T_2_ (indication for CPAP: RDI ≥ 15/hour with or without symptoms, or RDI 5–14/hour accompanied by comorbidities such as diabetes mellitus or symptoms as fatigue) (70) since their OSAS was completely resolved or was only mild. Cessation of CPAP therapy reduces costs and discomfort for patients. In an earlier study, OSAS severity decreased after achieving disease control, but not to an extent that CPAP therapy became redundant (17). In that study, the RDI decreased from 31/hour at baseline to 24/hour 6 months after achievement of disease control, which was insufficient to eliminate the indication for CPAP. However, they included a more heterogeneous group of patients with a male predominance and a higher BMI compared to our study. In addition, despite a clear indication, their patients were not treated with CPAP in the interval between the PSGs. In our study, a stronger reduction in the RDI was observed and the majority of our patients therefore lost their indication for CPAP therapy.

A limitation of our study is the relatively small cohort of patients, which makes it difficult to perform subgroup analyses to elucidate factors that influence prevalence and severity of OSAS in acromegaly. However, given the low incidence of acromegaly and the scarcity of treatment-naive patients, it is extremely hard to obtain larger and more homogeneous groups of patients. Nonetheless, our results were highly consistent. In addition, since we performed the second PSG 1 year after diagnosis and most patients underwent both SSA treatment and EETA during this period, it is not possible to differentiate between the distinct effects of those treatments, nor to identify the moment at which the largest changes take place in more detail.

In addition, we did not measure waist circumference, which is a surrogate marker of intra-abdominal fat mass. It has been previously reported that visceral fat mass increases during acromegaly treatment (71, 72), and that increased intra-abdominal fat mass is associated with OSAS (73). Waist circumference was also reported to be a risk factor for OSAS in acromegaly patients (12). Likewise, a larger neck circumference has been reported to be an OSAS risk factor, both in acromegaly patients (9, 74) and in the general population (75, 76). The relation between these easily accessible parameters and sleep parameters/OSAS is highly relevant to address in future studies, and might facilitate OSAS risk prediction in acromegaly patients, alone or in combination with other risk factors as male sex and BMI. This can eventually obviate PSG in acromegaly patients who are unlikely to have OSAS, and thereby contribute to cost-effective health care.

It is possible that long-term use of CPAP has an effect on some outcome measures. Previous reports suggested that the RDI/AHI could be lower in the period directly after withdrawal of CPAP: the so-called washout effect. Therefore, a wash-out period, during which the patient does not use CPAP, is advised prior to a PSG. However, solid data regarding the required duration of this washout period are not available. In a 2015 review article, a washout period of approximately 1 week was suggested, since 1 study reported a more pronounced increase in the RDI 7 days after discontinuation of CPAP compared with 1 day after discontinuation of CPAP (77). Other studies did not confirm this finding and reported an increase in RDI after 1 night without CPAP and a stable RDI during the week thereafter (77, 78). For practical reasons and in order to minimize discomfort for our patients, the participants in our study stopped CPAP therapy 3 nights prior to each PSG. Therefore, it is unlikely, but not impossible, that CPAP therapy influenced our outcomes.

Last, during the conductance of this study, the AASM released new guidelines regarding treatment of OSAS with CPAP therapy (79). We used the previous criteria throughout this study in order to be able to determine changes within individuals and to be able to compare groups of patients. When using the latest criteria, CPAP therapy would have been recommended in 3 additional female patients with asymptomatic mild OSAS and hypertension. Of these patients, 1 patient discontinued the study right after T_0_, 1 patient was cured from OSAS at T_2_, and the last patient had mild asymptomatic OSAS at T_1_ and did not undergo PSG at T_2_.

Given the high prevalence of OSAS in acromegaly and the high rate of improvement during treatment, we strongly recommend performing a PSG at diagnosis and treatment of OSAS when present. Furthermore, particularly in the case of IGF-1 normalization, we advise repeating the PSG 1 to 1.5 years later, since, based on the results of this study, there is a high chance for improvement of OSAS after decline or normalization of IGF-1 levels. If OSAS resolves, PSGs and CPAP therapy are no longer indicated, which leads to cost reduction and less discomfort for patients. Given the strong relation between the RDI and IGF-1 levels, it is not expected that patients in stable remission of acromegaly would develop OSAS, as long as other risk factors for development of OSAS are not present. This approach likely prevents or mitigates OSAS-related (cardiovascular) morbidity in acromegaly, functional limitations, and loss of quality of life.

In conclusion, OSAS resolved or improved in all acromegaly patients who were surgically or biochemically controlled, with the largest changes taking place during the first year of treatment. The improvement of OSAS parameters is related to the decrease in IGF-1 levels; this represents another clinically relevant reason for stringently aiming towards normalization of IGF-1 in patients with acromegaly. Based on our results, we recommend performing sleep studies in acromegaly patients.

## Abbreviations

AASM: American Academy of Sleep Medicine
AHI: Apnea Hypopnea Index
BMI: body mass index
CPAP: continuous positive airway pressure
CVD: cardiovascular disease
DA: dopamine agonist
EETA: endoscopic endonasal transsphenoidal adenomectomy
ESS: Epworth Sleepiness Scale
GH: growth hormone
IGF-1: insulin-like growth factor 1
ITT: insulin tolerance test
LSaO2: lowest oxygen saturation
MRI: magnetic resonance imaging
ODI: oxygen desaturation index
oGTT: oral glucose tolerance test
OSAS: obstructive sleep apnea syndrome
PEGV: pegvisomant
PSG: polysomnography
RDI: respiratory disturbance index
RERAs: respiratory effort–related arousals
SAS: sleep apnea syndrome
SSA: somatostatin receptor analogue

## Additional Information

***Disclosure Summary:*** The authors declare that there is no conflict of interest that could be perceived as prejudicing the impartiality of the research reported.

***Data Availability:*** The data sets generated during and/or analyzed during the current study are not publicly available but are available from the corresponding author on reasonable request.

## References

[1] Katznelson L, Laws ER Jr, Melmed S, et al. Endocrine Society . Acromegaly: an endocrine society clinical practice guideline. J Clin Endocrinol Metab. 2014;99(11):3933–3951. 25356808 10.1210/jc.2014-2700

[2] Colao A, Ferone D, Marzullo P, Lombardi G. Systemic complications of acromegaly: epidemiology, pathogenesis, and management. Endocr Rev. 2004;25(1):102–152. 14769829 10.1210/er.2002-0022

[3] Castellani C, Francia G, Dalle Carbonare L, et al. Morphological study of upper airways and long-term follow-up of obstructive sleep apnea syndrome in acromegalic patients. Endocrine. 2016;51(2):308–316. 26093846 10.1007/s12020-015-0659-x

[4] Dostalova S, Sonka K, Smahel Z, Weiss V, Marek J, Horinek D. Craniofacial abnormalities and their relevance for sleep apnoea syndrome aetiopathogenesis in acromegaly. Eur J Endocrinol. 2001;144(5):491–497. 11331215 10.1530/eje.0.1440491

[5] Vaessen TJ, Overeem S, Sitskoorn MM. Cognitive complaints in obstructive sleep apnea. Sleep Med Rev. 2015;19:51–58. 24846772 10.1016/j.smrv.2014.03.008

[6] Epstein LJ, Kristo D, Strollo PJ Jr, et al. Adult Obstructive Sleep Apnea Task Force of the American Academy of Sleep Medicine. Clinical guideline for the evaluation, management and long-term care of obstructive sleep apnea in adults. J Clin Sleep Med. 2009;5(3):263–276. 19960649 PMC2699173

[7] Herrmann BL, Wessendorf TE, Ajaj W, Kahlke S, Teschler H, Mann K. Effects of octreotide on sleep apnoea and tongue volume (magnetic resonance imaging) in patients with acromegaly. Eur J Endocrinol. 2004;151(3):309–315. 15362959 10.1530/eje.0.1510309

[8] Al Lawati NM, Patel SR, Ayas NT. Epidemiology, risk factors, and consequences of obstructive sleep apnea and short sleep duration. Prog Cardiovasc Dis. 2009;51(4):285–293. 19110130 10.1016/j.pcad.2008.08.001

[9] Turan O, Akinci B, Ikiz AO, et al. Airway and sleep disorders in patients with acromegaly. Clin Respir J. 2018;12(3):1003–1010. 28224726 10.1111/crj.12618

[10] Uyar M, Davutoglu V. An update on cardiovascular effects of obstructive sleep apnoea syndrome. Postgrad Med J. 2016;92(1091):540–544. 27317753 10.1136/postgradmedj-2016-134093

[11] Melmed S . Acromegaly pathogenesis and treatment. J Clin Invest. 2009;119(11):3189–3202. 19884662 10.1172/JCI39375 PMC2769196

[12] Vouzouneraki K, Franklin KA, Forsgren M, et al. Temporal relationship of sleep apnea and acromegaly: a nationwide study. Endocrine. 2018;62(2):456–463. 30066288 10.1007/s12020-018-1694-1 PMC6208862

[13] Hernández-Gordillo D, Ortega-Gómez Mdel R, Galicia-Polo L, et al. Sleep apnea in patients with acromegaly. Frequency, characterization and positive pressure titration. Open Respir Med J. 2012;6:28–33. 22754597 10.2174/1874306401206010028 PMC3386499

[14] Annamalai AK, Webb A, Kandasamy N, et al. A comprehensive study of clinical, biochemical, radiological, vascular, cardiac, and sleep parameters in an unselected cohort of patients with acromegaly undergoing presurgical somatostatin receptor ligand therapy. J Clin Endocrinol Metab. 2013;98(3):1040–1050. 23393175 10.1210/jc.2012-3072

[15] Chemla D, Attal P, Maione L, et al. Impact of successful treatment of acromegaly on overnight heart rate variability and sleep apnea. J Clin Endocrinol Metab. 2014;99(8):2925–2931. 24780045 10.1210/jc.2013-4288

[16] Sze L, Schmid C, Bloch KE, Bernays R, Brändle M. Effect of transsphenoidal surgery on sleep apnoea in acromegaly. Eur J Endocrinol. 2007;156(3):321–329. 17322492 10.1530/eje.1.02340

[17] Akkoyunlu ME, Ilhan MM, Bayram M, et al. Does hormonal control obviate positive airway pressure therapy in acromegaly with sleep-disordered breathing? Respir Med. 2013;107(11):1803–1809. 24074722 10.1016/j.rmed.2013.08.043

[18] Davi’ MV, Dalle Carbonare L, Giustina A, et al. Sleep apnoea syndrome is highly prevalent in acromegaly and only partially reversible after biochemical control of the disease. Eur J Endocrinol. 2008;159(5):533–540. 18765561 10.1530/EJE-08-0442

[19] Roemmler J, Gutt B, Fischer R, et al. Elevated incidence of sleep apnoea in acromegaly-correlation to disease activity. Sleep Breath. 2012;16(4):1247–1253. 22241151 10.1007/s11325-011-0641-7

[20] Vannucci L, Luciani P, Gagliardi E, et al. Assessment of sleep apnea syndrome in treated acromegalic patients and correlation of its severity with clinical and laboratory parameters. J Endocrinol Invest. 2013;36(4):237–242. 22776855 10.3275/8513

[21] Giustina A, Chanson P, Bronstein MD, et al. Acromegaly Consensus Group. A consensus on criteria for cure of acromegaly. J Clin Endocrinol Metab. 2010;95(7):3141–3148. 20410227 10.1210/jc.2009-2670

[22] Arlt W, Allolio B. Adrenal insufficiency. Lancet. 2003;361(9372):1881–1893. 12788587 10.1016/S0140-6736(03)13492-7

[23] Whelton PK, Carey RM, Aronow WS, et al. 2017 ACC/AHA/AAPA/ABC/ACPM/AGS/APhA/ASH/ASPC/NMA/PCNA guideline for the prevention, detection, evaluation, and management of high blood pressure in adults: a report of the American College of Cardiology/American Heart Association task force on clinical practice guidelines. J Am Coll Cardiol. 2018;71(19):e127–e248. 29146535 10.1016/j.jacc.2017.11.006

[24] American Diabetes A. 2. Classification and diagnosis of diabetes: standards of medical care in diabetes-2019. Diabetes Care. 2019;42(Suppl 1):S13–S28. 30559228 10.2337/dc19-S002

[25] Grundy SM, Stone NJ, Bailey AL, et al. 2018 AHA/ACC/AACVPR/AAPA/ABC/ACPM/ADA/AGS/APhA/ASPC/NLA/PCNA guideline on the management of blood cholesterol: executive summary: a report of the American College of Cardiology/American Heart Association task force on clinical practice guidelines. J Am Coll Cardiol. 2019;73(24):3168–3209. 30423391 10.1016/j.jacc.2018.11.002

[26] Berry RB, Budhiraja R, Gottlieb DJ, et al. American Academy of Sleep Medicine. Rules for scoring respiratory events in sleep: update of the 2007 AASM manual for the scoring of sleep and associated events. deliberations of the sleep apnea definitions task force of the American Academy of Sleep Medicine. J Clin Sleep Med. 2012;8(5):597–619. 23066376 10.5664/jcsm.2172 PMC3459210

[27] Johns MW . A new method for measuring daytime sleepiness: the Epworth sleepiness scale. Sleep. 1991;14(6):540–545. 1798888 10.1093/sleep/14.6.540

[28] Johns M, Hocking B. Daytime sleepiness and sleep habits of Australian workers. Sleep. 1997;20(10):844–849. 9415943 10.1093/sleep/20.10.844

[29] Bland JM, Altman DG. Calculating correlation coefficients with repeated observations: Part 1–correlation within subjects. BMJ. 1995;310(6977):446. 7873953 10.1136/bmj.310.6977.446 PMC2548822

[30] Roerink S, Marsman D, van Bon A, Netea-Maier R. A missed diagnosis of acromegaly during a female-to-male gender transition. Arch Sex Behav. 2014;43(6):1199–1201. 24867179 10.1007/s10508-014-0309-z

[31] Attal P, Chanson P. Endocrine aspects of obstructive sleep apnea. J Clin Endocrinol Metab. 2010;95(2):483–495. 20061419 10.1210/jc.2009-1912

[32] Grunstein RR, Ho KK, Sullivan CE. Effect of octreotide, a somatostatin analog, on sleep apnea in patients with acromegaly. Ann Intern Med. 1994;121(7):478–483. 8067645 10.7326/0003-4819-121-7-199410010-00002

[33] Ip MS, Tan KC, Peh WC, Lam KS. Effect of Sandostatin LAR on sleep apnoea in acromegaly: correlation with computerized tomographic cephalometry and hormonal activity. Clin Endocrinol (Oxf). 2001;55(4):477–483. 11678830 10.1046/j.1365-2265.2001.01358.x

[34] Mickelson SA, Rosenthal LD, Rock JP, Senior BA, Friduss ME. Obstructive sleep apnea syndrome and acromegaly. Otolaryngol Head Neck Surg. 1994;111(1):25–30. 8028937 10.1177/019459989411100107

[35] Grunstein RR, Ho KY, Sullivan CE. Sleep apnea in acromegaly. Ann Intern Med. 1991;115(7):527–532. 1883121 10.7326/0003-4819-115-7-527

[36] Isono S, Saeki N, Tanaka A, Nishino T. Collapsibility of passive pharynx in patients with acromegaly. Am J Respir Crit Care Med. 1999;160(1):64–68. 10390381 10.1164/ajrccm.160.1.9806054

[37] Fatti LM, Scacchi M, Pincelli AI, Lavezzi E, Cavagnini F. Prevalence and pathogenesis of sleep apnea and lung disease in acromegaly. Pituitary. 2001;4(4):259–262. 12501976 10.1023/a:1020702631793

[38] Wagenmakers MA, Roerink SH, Maal TJ, et al. Three-dimensional facial analysis in acromegaly: a novel tool to quantify craniofacial characteristics after long-term remission. Pituitary. 2015;18(1):126–134. 24706165 10.1007/s11102-014-0565-x

[39] Guo X, Gao L, Zhao Y, et al. Characteristics of the upper respiratory tract in patients with acromegaly and correlations with obstructive sleep apnoea/hypopnea syndrome. Sleep Med. 2018;48:27–34. 29852361 10.1016/j.sleep.2018.04.011

[40] Üçler R, Aslan M, Atmaca M, Alay M, Ademoğlu EN, Gülşen I. Evaluation of blood neutrophil to lymphocyte and platelet to lymphocyte ratios according to plasma glucose status and serum insulin-like growth factor 1 levels in patients with acromegaly. Hum Exp Toxicol. 2016;35(6):608–612. 26224042 10.1177/0960327115597313

[41] Arikan S, Bahceci M, Tuzcu A, Gokalp D. Serum tumour necrosis factor-alpha and interleukin-8 levels in acromegalic patients: acromegaly may be associated with moderate inflammation. Clin Endocrinol (Oxf). 2009;70(3):498–499. 18673463 10.1111/j.1365-2265.2008.03362.x

[42] Olarescu NC, Ueland T, Godang K, Lindberg-Larsen R, Jørgensen JO, Bollerslev J. Inflammatory adipokines contribute to insulin resistance in active acromegaly and respond differently to different treatment modalities. Eur J Endocrinol. 2014;170(1):39–48. 24092547 10.1530/EJE-13-0523

[43] Kohler M . Why should we care about upper airway inflammation in obstructive sleep apnoea? Eur Respir J. 2016;48(4):982–983. 27694409 10.1183/13993003.01248-2016

[44] Berg C, Wessendorf TE, Mortsch F, et al. Influence of disease control with pegvisomant on sleep apnoea and tongue volume in patients with active acromegaly. Eur J Endocrinol. 2009;161(6):829–835. 19773369 10.1530/EJE-09-0694

[45] Turhan M, Bostanci A, Bozkurt S. Estimation of cardiovascular disease from polysomnographic parameters in sleep-disordered breathing. Eur Arch Otorhinolaryngol. 2016;273(12): 4585–4593. 27363409 10.1007/s00405-016-4176-1

[46] Kendzerska T, Gershon AS, Hawker G, Leung RS, Tomlinson G. Obstructive sleep apnea and risk of cardiovascular events and all-cause mortality: a decade-long historical cohort study. PLoS Med. 2014;11(2):e1001599. 24503600 10.1371/journal.pmed.1001599 PMC3913558

[47] van der Klaauw AA, Pereira AM, van Kralingen KW, Rabe KF, Romijn JA. Somatostatin analog treatment is associated with an increased sleep latency in patients with long-term biochemical remission of acromegaly. Growth Horm IGF Res. 2008;18(5):446–453. 18502671 10.1016/j.ghir.2008.04.001

[48] Doff MH, Hoekema A, Wijkstra PJ, et al. Oral appliance versus continuous positive airway pressure in obstructive sleep apnea syndrome: a 2-year follow-up. Sleep. 2013;36(9):1289–1296. 23997361 10.5665/sleep.2948 PMC3738037

[49] Kapur VK, Auckley DH, Chowdhuri S, et al. Clinical practice guideline for diagnostic testing for adult obstructive sleep apnea: an American Academy of Sleep Medicine clinical practice guideline. J Clin Sleep Med. 2017;13(3):479–504. 28162150 10.5664/jcsm.6506 PMC5337595

[50] Pekkarinen T, Partinen M, Pelkonen R, Iivanainen M. Sleep apnoea and daytime sleepiness in acromegaly: relationship to endocrinological factors. Clin Endocrinol (Oxf). 1987;27(6):649–654. 3455370 10.1111/j.1365-2265.1987.tb02947.x

[51] van Haute FR, Taboada GF, Corrêa LL, et al. Prevalence of sleep apnea and metabolic abnormalities in patients with acromegaly and analysis of cephalometric parameters by magnetic resonance imaging. Eur J Endocrinol. 2008;158(4):459–465. 18362291 10.1530/EJE-07-0753

[52] Young T, Skatrud J, Peppard PE. Risk factors for obstructive sleep apnea in adults. JAMA. 2004;291(16):2013–2016. 15113821 10.1001/jama.291.16.2013

[53] Weiss V, Sonka K, Pretl M, et al. Prevalence of the sleep apnea syndrome in acromegaly population. J Endocrinol Invest. 2000;23(8):515–519. 11021767 10.1007/BF03343767

[54] Burgers AM, Kokshoorn NE, Pereira AM, et al. Low incidence of adrenal insufficiency after transsphenoidal surgery in patients with acromegaly: a long-term follow-up study. J Clin Endocrinol Metab. 2011;96(7):E1163–E1170. 21470989 10.1210/jc.2010-2673

[55] Wagenmakers MA, Netea-Maier RT, van Lindert EJ, Pieters GF, Grotenhuis AJ, Hermus AR. Results of endoscopic transsphenoidal pituitary surgery in 40 patients with a growth hormone-secreting macroadenoma. Acta Neurochir (Wien). 2011;153(7):1391–1399. 21347581 10.1007/s00701-011-0959-8 PMC3111724

[56] Kritikou I, Basta M, Vgontzas AN, et al. Sleep apnoea and the hypothalamic-pituitary-adrenal axis in men and women: effects of continuous positive airway pressure. Eur Respir J. 2016;47(2):531–540. 26541531 10.1183/13993003.00319-2015 PMC7090379

[57] Karaca Z, Ismailogullari S, Korkmaz S, et al. Obstructive sleep apnoea syndrome is associated with relative hypocortisolemia and decreased hypothalamo-pituitary-adrenal axis response to 1 and 250μg ACTH and glucagon stimulation tests. Sleep Med. 2013;14(2):160–164. 23218531 10.1016/j.sleep.2012.10.013

[58] Tomfohr LM, Edwards KM, Dimsdale JE. Is obstructive sleep apnea associated with cortisol levels? A systematic review of the research evidence. Sleep Med Rev. 2012;16(3):243–249. 21803621 10.1016/j.smrv.2011.05.003 PMC3242892

[59] Winkelman JW, Goldman H, Piscatelli N, Lukas SE, Dorsey CM, Cunningham S. Are thyroid function tests necessary in patients with suspected sleep apnea? Sleep. 1996;19(10):790–793. 9085487 10.1093/sleep/19.10.790

[60] Bahammam SA, Sharif MM, Jammah AA, Bahammam AS. Prevalence of thyroid disease in patients with obstructive sleep apnea. Respir Med. 2011;105(11):1755–1760. 21820299 10.1016/j.rmed.2011.07.007

[61] Bielicki P, Przybyłowski T, Kumor M, Barnaś M, Wiercioch M, Chazan R. Thyroid hormone levels and TSH activity in patients with obstructive sleep apnea syndrome. Adv Exp Med Biol. 2016;878:67–71. 26542600 10.1007/5584_2015_180

[62] Ozcan KM, Selcuk A, Ozcan I, et al. Incidence of hypothyroidism and its correlation with polysomnography findings in obstructive sleep apnea. Eur Arch Otorhinolaryngol. 2014;271(11):2937–2941. 24609648 10.1007/s00405-014-2962-1

[63] Lozo T, Komnenov D, Badr MS, Mateika JH. Sex differences in sleep disordered breathing in adults. Respir Physiol Neurobiol. 2017;245:65–75. 27836648 10.1016/j.resp.2016.11.001

[64] Barrett-Connor E, Dam TT, Stone K, Harrison SL, Redline S, Orwoll E; Osteoporotic Fractures in Men Study Group. The association of testosterone levels with overall sleep quality, sleep architecture, and sleep-disordered breathing. J Clin Endocrinol Metab. 2008;93(7):2602–2609. 18413429 10.1210/jc.2007-2622 PMC2453053

[65] Liu PY, Swerdloff RS, Veldhuis JD. Clinical review 171: the rationale, efficacy and safety of androgen therapy in older men: future research and current practice recommendations. J Clin Endocrinol Metab. 2004;89(10):4789–4796. 15472164 10.1210/jc.2004-0807

[66] Katznelson L, Kleinberg D, Vance ML, et al. Hypogonadism in patients with acromegaly: data from the multi-centre acromegaly registry pilot study. Clin Endocrinol (Oxf). 2001;54(2):183–188. 11207632 10.1046/j.1365-2265.2001.01214.x

[67] Grynberg M, Salenave S, Young J, Chanson P. Female gonadal function before and after treatment of acromegaly. J Clin Endocrinol Metab. 2010;95(10):4518–4525. 20660045 10.1210/jc.2009-2815

[68] Jane JA Jr, Starke RM, Elzoghby MA, et al. Endoscopic transsphenoidal surgery for acromegaly: remission using modern criteria, complications, and predictors of outcome. J Clin Endocrinol Metab. 2011;96(9):2732–2740. 21715544 10.1210/jc.2011-0554

[69] Cozzi R, Montini M, Attanasio R, et al. Primary treatment of acromegaly with octreotide LAR: a long-term (up to nine years) prospective study of its efficacy in the control of disease activity and tumor shrinkage. J Clin Endocrinol Metab. 2006;91(4):1397–1403. 16449332 10.1210/jc.2005-2347

[70] Kushida CA, Littner MR, Hirshkowitz M, et al. American Academy of Sleep Medicine. Practice parameters for the use of continuous and bilevel positive airway pressure devices to treat adult patients with sleep-related breathing disorders. Sleep. 2006;29(3):375–380. 16553024 10.1093/sleep/29.3.375

[71] Reyes-Vidal CM, Mojahed H, Shen W, et al. Adipose tissue redistribution and ectopic lipid deposition in active acromegaly and effects of surgical treatment. J Clin Endocrinol Metab. 2015;jc20151917. 10.1210/jc.2015-1917 PMC4524994 26037515

[72] Guo X, Gao L, Shi X, et al. Pre- and postoperative body composition and metabolic characteristics in patients with acromegaly: a prospective study. Int J Endocrinol. 2018;2018:4125013. 29531529 10.1155/2018/4125013 PMC5817290

[73] Vgontzas AN, Bixler EO, Chrousos GP. Metabolic disturbances in obesity versus sleep apnoea: the importance of visceral obesity and insulin resistance. J Intern Med. 2003;254(1):32–44. 12823641 10.1046/j.1365-2796.2003.01177.x

[74] Rosenow F, Reuter S, Deuss U, et al. Sleep apnoea in treated acromegaly: relative frequency and predisposing factors. Clin Endocrinol (Oxf). 1996;45(5):563–569. 8977753 10.1046/j.1365-2265.1996.00852.x

[75] Hoffstein V, Mateika S. Differences in abdominal and neck circumferences in patients with and without obstructive sleep apnoea. Eur Respir J. 1992;5(4):377–381. 1563498

[76] Davies RJ, Ali NJ, Stradling JR. Neck circumference and other clinical features in the diagnosis of the obstructive sleep apnoea syndrome. Thorax. 1992;47(2):101–105. 1549815 10.1136/thx.47.2.101 PMC463582

[77] Vroegop AV, Smithuis JW, Benoist LB, Vanderveken OM, de Vries N. CPAP washout prior to reevaluation polysomnography: a sleep surgeon’s perspective. Sleep Breath. 2015;19(2): 433–439. 25487311 10.1007/s11325-014-1086-6

[78] Yang Q, Phillips CL, Melehan KL, Rogers NL, Seale JP, Grunstein RR. Effects of short-term CPAP withdrawal on neurobehavioral performance in patients with obstructive sleep apnea. Sleep. 2006;29(4):545–552. 16676788 10.1093/sleep/29.4.545

[79] Patil SP, Ayappa IA, Caples SM, Kimoff RJ, Patel SR, Harrod CG. Treatment of adult obstructive sleep apnea with positive airway pressure: an American Academy of Sleep Medicine clinical practice guideline. J Clin Sleep Med. 2019;15(2):335–343. 30736887 10.5664/jcsm.7640 PMC6374094

